# Immunohistochemical Characterisation of Cell-Type Specific Expression of CK1δ in Various Tissues of Young Adult BALB/c Mice

**DOI:** 10.1371/journal.pone.0004174

**Published:** 2009-01-12

**Authors:** Jürgen Löhler, Heidrun Hirner, Bernhard Schmidt, Klaus Kramer, Dietmar Fischer, Dietmar R. Thal, Frank Leithäuser, Uwe Knippschild

**Affiliations:** 1 Molecular Pathology Group, Heinrich-Pette-Institute for Experimental Immunology and Virology, University Hamburg, Hamburg, Germany; 2 Department of General-, Visceral- and Transplantation Surgery, University of Ulm, Ulm, Germany; 3 Department of Experimental Neurology, University of Ulm, Ulm, Germany; 4 Laboratory of Neuropathology, Institute of Pathology, University of Ulm, Ulm, Germany; 5 Department of Pathology, University of Ulm, Ulm, Germany; National Institutes of Health, United States of America

## Abstract

**Background:**

Casein kinase 1 delta (CK1δ) phosphorylates many key proteins playing important roles in such biological processes as cell growth, differentiation, apoptosis, circadian rhythm and vesicle transport. Furthermore, deregulation of CK1δ has been linked to neurodegenerative diseases and cancer. In this study, the cell specific distribution of CK1δ in various tissues and organs of young adult BALB/c mice was analysed by immunohistochemistry.

**Methodology/Principal Findings:**

Immunohistochemical staining of CK1δ was performed using three different antibodies against CK1δ. A high expression of CK1δ was found in a variety of tissues and organ systems and in several cell types of endodermal, mesodermal and ectodermal origin.

**Conclusions:**

These results give an overview of the cell-type specific expression of CK1δ in different organs under normal conditions. Thus, they provide evidence for possible cell-type specific functions of CK1δ, where CK1δ can interact with and modulate the activity of key regulator proteins by site directed phosphorylation. Furthermore, they provide the basis for future analyses of CK1δ in these tissues.

## Introduction

The mammalian members of the CK1 (formerly casein kinase 1) family, namely CK1α, β, γ_1–3_, δ and ε and their various splice variants, are ubiquitiously expressed. They are highly conserved within their kinase domains, but they significantly differ in lengths and primary structures of their regulatory N- and C-terminal non-catalytic domains (reviewed in [1], [2]). Within the cell CK1 isoforms are found in the nucleus, the cytoplasm and at the plasma membrane (reviewed in [1], [2]). They are able to phosphorylate many different substrates bearing either a canonical or non-canonical consensus sequence [1], [3]–[5]. As a result, they can modulate the activity of key regulator proteins involved in biological processes such as cell differentiation [6]–[11], cell proliferation, apoptosis [12]–[16], circadian rhythm [17], chromosome segregation [18]–[21], and vesicle transport [19], [20], [22]. Considering the importance of CK1-mediated signals, it is obvious that mutations and/or changes in the activity of CK1 isoforms, especially of CK1δ and ε, or mutations of CK1 specific phosphorylation sites within their substrates can be pathogenic, leading to neurodegenerative diseases [23]–[26], sleeping disorders [27]–[30], and/or cancer [2], [31]–[36].

Recently, interest has increased to clarify the physiological functions of CK1δ. Previous studies on mRNA and protein level revealed an ubiquitous distribution of CK1δ [35], [37], [38]. Furthermore, differences in the activity of CK1δ in tissues with similar expression levels indicate that posttranslational modifications, especially site-specific phosphorylation, play an important role in regulating the activity of CK1δ ([2] and references therein, [39]). In addition, it has been suggested that CK1δ plays an important role in regulating several aspects of lymphocyte physiology [35].

In this report we use immunohistrochemistry (IHC) to determine the tissue and cell-type specific distribution of CK1δ in healthy mice. Providing an anatomical fundament, our results may contribute to better understanding the possible cell-type specific functions of CK1δ under physiological conditions.

## Results

### Fixation and immunolabelling

**Figure 1:**
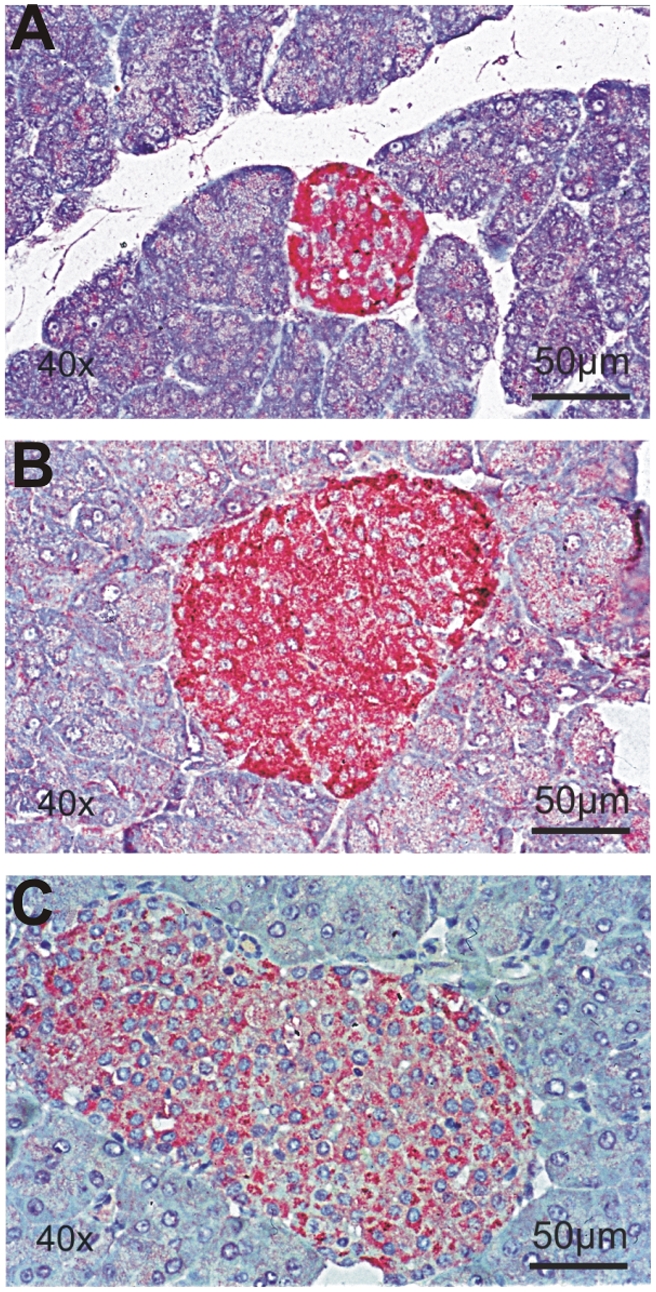
Specificity of the anti CK1δ polyclonal rabbit serum NC10. An immunoabsorption test for NC10 was used to show its specificity in immunohistochemistry (IHC). Immersion fixation with acetic formalin, alkaline phosphatase reaction, dye: newfuchsin. IHC was performed on paraffin-embedded pancreatic tissue of a 5 week old BALB/c mouse using NC10 (A) or NC10 preincubated with either a control peptide (the p53 specific peptide MEESQSDISLELGGC, 0.1 µg (B)) or with the specific blocking peptide used for immunisation of rabbits (CGDMASLRLHAARQGARC, 0.1 µg) for 3 h at 4°C (C). The results indicate that the antigenic peptide, but not the control peptide competively inhibits CK1δ binding. Magnification: 400×.

**Figure 2:**
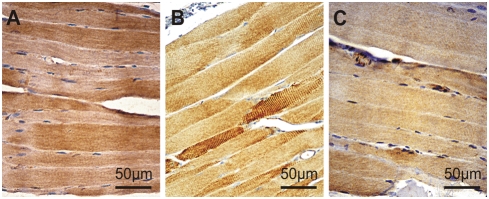
Immunohistochemical detection of CK1δ in skeletal muscles of a six week old BALB/c mouse. Longitudinal section. Perfusion fixation with Bouin. Peroxidase reaction, dye: DAB. A similar CK1δ staining pattern of the myofibrils was detected independent of the antibody (NC10, 108, or ab10877). Specific antibody binding was visualised by the peroxidase reaction using DAB as substrate-chromogen. (A) NC10, immersion fixation with acid formalin; (B) 108, perfusion fixation with Bouin; (C): ab10877 (Abcam), immersion fixation with acid formalin. Magnification 100×. Using three different CK1δ specific antibodies only minor variations in the CK1δ staining pattern were observed. These could be explained by alterations in the phosphorylation status of CK1δ influencing the disposability of the particular antigenous epitope and/or by differences in the recognition of CK1δ splice variants being differently expressed in those cases.

Previously, we have shown that CK1δ protein is ubiquitously expressed in mouse tissues and organs. Furthermore, differences in protein and functional activity levels have been detected [35]. In this study, the cell-type specific expression patterns of CK1δ in mouse tissues were further examined by IHC. Since CK1δ is immediately induced upon cellular stress [40], it is crucial to obtain an efficient and fast fixation of the tissue. To optimise the immunohistochemical detection of CK1δ the effects of different fixations, fixatives, blocking solutions, and antigen demasking procedures were tested (see Tables 1–3). In addition, the suitability and specificity of three CK1δ specific antibodies (NC10, 108, ab10877) were characterised (Figures 1 and 2, see Material and Methods section, Behrend and co-workers [19], and Stöter and co-workers [36]).

**Table 1:** Effect of different fixation agents and fixation methods on antigen detection.

fixation solution	antigen preservation	morphology	number of animals
**perfusion fixation**			**8**
• Bouin solution	+ +(+)	+ + +	2
• Acid formalin (4%)	+ + (+)	+ + +	3
• Neutral buffered formalin (10%)	+ +	+ + +	3
**immersion fixation**			**24**
• Bouin solution	+ +	+ + (+)	2
• acid formalin (4%)	+ +	+ + (+)	6
• neutral buffered formalin (10%)	+ (+)	+ + (+)	6
• Carnoy-fixation	− −	+ +	2
• Zinc-fixation	+ (+)	−	2
• Glyo Fixx (Shandon)	+	+ +	2
• Zinc Formal-Fixx (Shandon)	(+)	+ +	2
• Notox Histological Fixative (Quartett)	−/+	+ +	2

To establish the immunohistochemical detection of CK1δ several fixation methods and fixatives had been used. Paraffin embedded tissue sections were immunostained for CK1δ using different fixation solutions and fixation methods. Comparison of the perfusion with the immersion fixation method revealed only slight regional differences in the preservation of CK1δ and in the morphology of the tissue especially when acidic formalin or Bouin's fluid were used as fixatives. − no effect on antigen detection or morphology; + weak effect on antigen detection or morphology; ++ medium effect on antigen detection or morphology ; +++ strong effect on antigen detection or morphology. (+) indicates intermediate effect, for instance ++(+) indicates intermediate level between medium and strong effect.

**Table 2 pone-0004174-t002:** Heat induced antigen demasking.

Demasking solution	Pab NC10	Pab 108	Pab ab10877
TUF (Target unmasking fluid) (Kreatech)			
• pH 5,70	+ + +	− to −/+	+
Citra Plus 10× (Biogenex)			
• pH 6,0	+ +	−/+	+ + +
		+ + + (with autoclave in microwave)	
AR-10 solution 10× (Biogenex)			
• pH 10,7	−/+	− to −/+	−/+
Tris-Puffer			
• pH 7,3	+	− to −/+	+

Sections of paraffin-embedded tissue were used to test the ability of various solutions to demask the antigen. The detection of CK1δ was evaluated by the specific colour intensity of the applied antibody using the scaling mentioned in the table: − no specific labelling, −/+ pale; + weak, + + moderate, + + + strong labelling.

**Table 3:** Effect of different blocking reagents on background reduction of CK1δ immunostained frozen sections.

**blocking solution**	**specific against**	**background reduction**
Levamisole	endogenous alkaline phosphatase	+ +
peroxidase-blocking reagens (DAKO)	endogenous peroxidase	+ +
Uni-Block (BioGenex)	endogenous biotin	+ + +
Avidin/Biotin Blocking Kit (Zymed)		+
**blocking solution**	**unspecific against**	**background reduction**
Aurion BSA-c, (Biotrend)	hydrophobic and electrostatic interactions	−
Power Block, Casein (BioGenex)		−
**anti- IgG F(ab′)_2_-fragment**		**background reduction**
• Anti-Rabbit IgG F(ab′)_2_ biotinylated (DAKO)		−

Frozen tissue sections were immunostained for CK1δ in the presence and absence of the indicated blocking reagens. − no effect of the blocking reagens; + low background reduction; ++ medium background reduction; +++ strong background reduction.

Different modes of fixation (i.e. immersion or perfusion) and various fixation solutions were compared with respect to antigen preservation, CK1δ staining intensity, and the preservation of tissue morphology in paraffin embedded tissues. As shown in Table 1 optimal antigen preservation was obtained with acetic acid formalin or Bouin's fixative. Comparison of perfusion with immersion fixation revealed only slight regional differences in the CK1δ staining intensity, and in the preservation of the morphology, especially when Bouin's solution, acid formalin or neutral buffered formalin were used as fixatives.

Different antigen retrievals were required to expose the particular CK1δ epitope recognised by each of the three different CK1δ specific antibodies in paraffin-embedded tissues. Best staining results for NC10 were obtained using TUF (Target Unmasking Fluid, the composition is not disclosed by Kreotech) at 90°C in a microwave oven, whereas the primary polyclonal antibodies PAb108 and PAb10877 showed better immunoreactivity by antigen retrieval in citric buffer in a microwave oven. PAb108 recognised CK1δ in paraffin-embedded tissues only when sections were heated in a pressure cooker inside a microwave oven (Table 2).

Staining of myelin sheets in neuronal tissues with diaminobencidine as a colour substrate gave inconsistent results, ranging from strongly positive to negative. Since diaminobencidine staining has been reported to label myelin sheets unspecifically [41], we considered the myelin staining as artificial.

When polyclonal CK1δ specific antibodies raised in rabbit (NC10, PAb108) or goat (ab10877) were compared, similar reactivities were detected in frozen and paraffin embedded tissues (see Figure 2 for an example). However, the tissue morphology was far better preserved in paraffin sections when compared to cryosections. Therefore, we decided to carry out all further immunostainings on paraffin embedded tissues.

### Cell specific tissue distribution of CK1δ

The CK1δ positive reactivities in various paraffin embedded tissues of young adult BALB/c mice obtained by immunohistochemistry are summarised in Table 4 and correspond well with our previous results obtained from Western blotting analyses [35].

**Table 4:** Localization and levels of CK1δ in 4 to 6 week old BALB/c mice.

**Thymus**	
Thymocytes outer cortex	++
Thymocytes inner cortex	−
Medulla	++/−
**Lymph node / MALT**	
Lymphoid blasts	++
lmyphocytes B-zone	+/−
lmyphocytes T-zone	++/−
**Spleen**	Refer to ref. [35]
**Muscle cells**	
Scelletal	++/−
Cardiac	++/−
Smooth	+/−
**Vascular endothelium**	
Arteries	++
Veins	+/−
Lymphatics	+/−
High endothelial venoles	+/−
**Mesothelial cells**	++
**Connective tissue**	
Adipocytes (univacuolar, polyvacuolar)	++
Fibroblasts/fibrocytes	+/−
Chondrocytes	−
Osteocytes, osteobasts	−
**Alimentary tract**	
Esophageal squamous epithelium, (basal cell layer)	−
Esophageal squamous epithelium (stratum granulosum, corneum)	+++
Stomach, chief cells	+++
Stomach, parietal cells	−
Stomach, mucous neck cells	+/+++
Intestine, absortive cells	++
Intestine, goblet cells	−
Intestine, paneth cells	−
**Urinary tract**	
Glomeruar endothelial cells	−
Mesangial cells	−
Podocytes	++
Bowman capsule	−
Tubulus epithelium	++/+++
Collecting duct epithelium	++/+++
Urothelium, basal cells	+
Urothelium, umbrella cells	−
**Female genital tract**	
Oocytes	−
Immature ovarian follicle, follicular epithelium	+++
Mature ovarian follicle, follicular epithelium	+
Theka cells	−
fallopian tube	+++
Uterus endometrium	+++
Stroma cells	−/++
**Male genital tract**	
Germinal cells, spermatogonia	++/+++
Spermatocytes	+
Sertoli cells	+
Leydig cells	+++
Epidymis epithelium	+++
Deferent duct epithelium	+++
Prostate gland	+++
Seminal vesicle	+++
**Salivary glands**	
Serous epithelial cells	++/+++
Mucous epithelial cells	−
Myoepithelium	−
Ductal epithelium	++/+++
**Pancreas**	
Exocrine gland, acinar cells	++
Exocrine gland, duct epithelium	+/−
Islets	+++/++
**Adrenal**	
Stratum granulosum	+
Stratum fasciculatum	+
x-zone	++
Medulla	++/+
Hypophysis	
Adenohypophysis	+++
Neurohypophysis	+++
Pars intermedia	+++
**Liver, gallbladder**	
Hepatocytes	++
Intrahepatic bile ducts	−
Gallbladder, extrahepatic bile ducts	−
**Thyroid gland**	
Follicular epithelium	++
**Eye**	
Conjunctival epithelium, basal cell layer	−
Conjunctival epithelium, superficial cell layer	+++
Sclera	−
Choroidea	−
Iris stroma	−
Iris posterior epithelial lining	++
Ciliary muscle	+
Ciliary epithelium	++
Lens capsule	−
Cuboidal epithelium of the lens	++
Lens nucleus	+++
*Retina*	
Choroid	−
Retinal pigment epithelium	+
Outer nuclear layer	−
Outer plexiform layer	+
Inner nuclear layer	++
Inner plexiform layer	+
Ganglion cell layer	+++
Nerve fibre layer	+
**Respiratory tract**	
Upper aerodigestive tract, squamous epithelium, basal cell layer	−
Upper aerodigestive tract, squamous epithelium, superficial cell layer	+++
Respiratory epithelium	+++
Type 1 pneumocytes	−
Type 2 pneumocytes	++
**Nervous system**	
**Peripheral nervous system**	
*Trigeminal ganglion*	
Nerve cell pericarium	++
Peripheral nerve fibre	−
*Spinal ganglion*	
Nerve cell pericarium	++
Peripheral nerve fibre	−
**Central nervous system**	
**Spinal cord**	
Gray matter – nerve cell pericaryon	+++
Gray matter neuropil	+
White matter	(+)
**Brain**	
*Nerve cell pericarium/neuropil*	
Cerebral neocortex	++/++
Hippocampus	++/++
Basal ganglia	+/−
Hypothalamus	+/−
Thalamus	+/−
Midbrain	+/−
Pons	+/−
Medulla oblongata	+/−
Purkinje cells/cerebellum	++
Granule cells/cerebellum	−/−
Dentate nucleus	+/−
White matter	(+)
**Skin and skin appendages**	
Epidermis (basal layer)	−
Epidermis (stratum granulosum, corneum)	+++
Sebacous gland	+++
Haderian gland	+++
Mammary gland, glandular epithelium	++
Mammary gland, myoepithelium	−

Intensity levels of the CK1δ specific staining were graded as − negative, + weak, ++ moderate, or +++ strong. Slash points to simultaneous expression of different intensities, e.g., −/++ indicates negative and moderately positive staining in one cell type. Staining results were nearly identical independent of the CK1δ specific antibody (NC10, 108, or ab10877) having been used.

#### Subcellular antigen distribution

In most immunoreactive cells CK1δ expression was restricted to the cytoplasm. Only in some cell populations, e.g. neurons of the hypothalamus, a nuclear immunoreactivity was observed.

#### Alimentary tract

**Figure 3:**
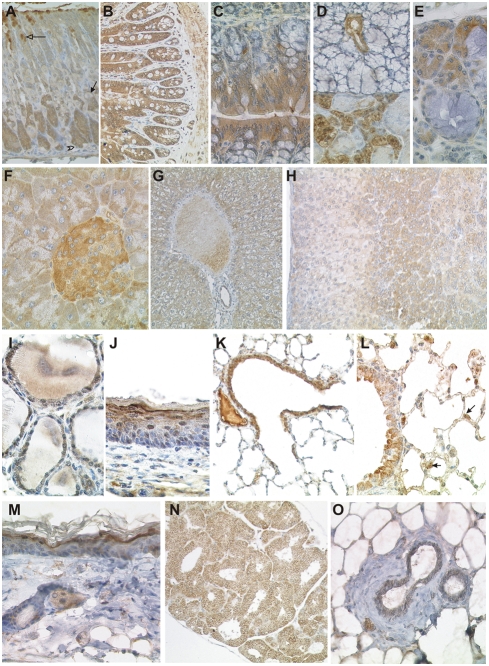
Immunohistochemical detection of CK1δ in the gastrointestinal tract, endocrine glands, lung, skin, and mammary gland. Fixative: acid formalin, fixation by immersion. Peroxidase reaction, dye: DAB. Immunohistochemical staining of CK1δ in the stomach (A); small intestinal (B); colon (C); large salivary gland (D); small salivary gland of the trachea (E); pancreas (F); liver (G); adrenal gland (H); thyroid gland (I); squamous epithelium of upper aerodigestive tract (J); lung (K, L), skin (M), harderian gland (N), and mammary gland (O). In (A), solid arrows point to parietal cells, open arrows to neck parietal cells and open arrowheads to chief cells. In figure 3L, solid arrows point to strong CK1δ positive type II pneumocytes. Magnification: 100× (A, B, K), 200× (G, H, J, N), 400× (C–F, I, L, M, O).

The epithelial cell layers of esophagus, stomach, and intestine (small intestine and colon) showed CK1δ immunoreactivity (Figure 3A, B, C). The stratified squamous epithelium lining the esophagus and the proximal part of the murine stomach displayed a gradual increase of CK1δ expression from the stratum basale, which was CK1δ negative compared to the strongly CK1δ positive stratum corneum. In the glandular part of the stomach, CK1δ was highly expressed in the chief cells located at the base of the gastric glands while parietal cells were negative. Mucous neck cells showed only faint CK1δ staining in the glandular isthmus (where they constitute the regenerative pool), but gained strong CK1δ positivity during their differentiation to mucous secreting cells found at the pits of the mucosa (Figure 3A). Absorptive epithelial cells of the intestinal villi and regenerative cells at the mucosal crypts were equally CK1δ positive (Figure 3B). In contrast, goblet cells and Paneth cells did not show any CK1δ staining. An analogous picture was seen in the colon, where absorptive and regenerative columnar cells expressed high amounts of CK1δ, while goblet cells were negative (Figure 3C).

#### Salivary glands and exocrine pancreas

The major salivary glands, i.e. the parotid, the sublingual and the submandibular glands, as well as the minor salivary glands of the oropharynx, the esophagus and the upper respiratory tract all expressed CK1δ, albeit to a different extent (Figure 3D, E). As a general pattern, mucous and myoepithelial cells were CK1δ negative, while serous epithelial cells and the salivary duct epithelium expressed intermediate to high levels of CK1δ. A somewhat diverging picture was seen in the exocrine part of the pancreas (Figure 3F). Here, the acinar cells showed moderate CK1δ positivity whereas the ductal epithelium was CK1δ negative to faintly positive.

#### Liver

In the liver (Figure 3G), a moderate CK1δ immunostaining was found in the hepatocytes. CK1δ expression in liver cells was evenly distributed throughout the hepatic lobule. The cytoplasm facing the sinusoids and the adjacent cell membrane seemed to be the most intensely stained part of the hepatocytes. This staining pattern might have obscured CK1δ expression by Kupffer cells, which was not detected. Expression of CK1δ could not be detected in the epithelium of the bile ducts and the gallbladder.

#### Endocrine organs

The thyroid follicular epithelium showed moderate CK1δ staining (Figure 3I). Pancreatic islets harboured a large population of strongly CK1δ positive cells and a smaller fraction of endocrine cells with moderate CK1δ expression (Figure 3F). In the adrenal cortex, low amounts of CK1δ were detected in the zona glomerulosa and the zona fasciculata while cells of the x-zone showed moderate CK1δ expression. Weak to moderate staining was detected in the adrenal medulla (Figure 3H). The expression pattern of CK1δ in the pituitary gland is described in the section “Peripheral and central nervous system”.

#### Respiratory tract

The stratified squamous epithelium of the upper aerodigestive tract displayed strong CK1δ expression in the basal cell layer and decreasing immunoreactivity towards the superficial layers (Figure 3J). Strong cytoplasmic CK1δ staining was observed in the ciliated epithelium of the trachea and the bronchial tree (Figure 3K). At the peripheral parts of the respiratory tract, CK1δ staining was lost upon transition from bronchioles to the respiratory spaces (Figure 3K, L). However, while flat alveolar epithelium cells (type I pneumocytes) were CK1δ negative, moderate CK1δ staining was detected in cuboidal type II pneumocytes (Figure 3L).

#### Skin and skin appendages

In accordance to the findings in the esophagus, the keratinised squamous epithelium of the epidermis displayed an increasing CK1δ expression from the weakly positive basal cell layer to the strongly CK1δ positive stratum granulosum (Figure 3M). Intensive CK1δ staining was also detected in the sebaceous glands of the hair follicle and in the harderian glands (Figure 3N). The mammary gland showed moderate levels of CK1δ expression in the columnar/cuboidal glandular epithelium while the myoepithelial cells remained negative (Figure 3O).

#### Urinary tract

**Figure 4:**
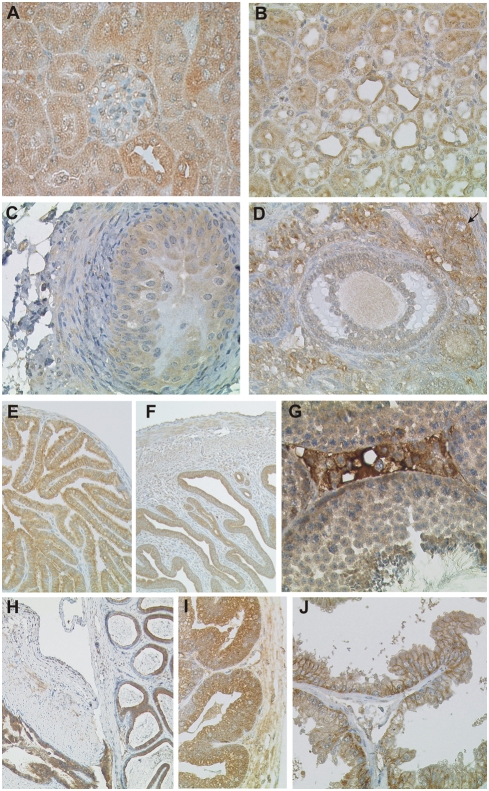
Immunohistochemical detection of CK1δ in the urogenital tract. Fixative: acid formalin, fixation by immersion. Peroxidase reaction, dye: DAB. The staining results of the CK1δ specific antiserum NC10 in organs of the urogenital tract are shown. (A, B) kidney; (C) ureter; (D) ovary; (E) fallopian tube; (F) uterus; (G) testis; (H) seminal duct and epididymis; (I) prostate; (J) seminal vesicle. The solid arrow indicates a primary follicle in D. Magnification: 100× (E, F, H), 200× (A, D, I, J), 400× (B, C, G), 640× (A).

In the renal glomerulum, moderate CK1δ expression was detected in large cells located predominantly in the capsule of gomerulae, most likely representing podocytes (Figure 4A). All other cell types of the glomerulum, including the parietal cells of Bowman's capsule, were CK1δ negative. The epithelial lining of the renal tubule and the collecting ducts displayed moderate to strong CK1δ immunoreactivity (Figure 4B). The so called transitional epithelium covering the efferent urinary tract from the renal pelvis to the urinary bladder was weakly CK1δ positive in the basal layer while the umbrella cells were CK1δ negative (Figure 4C and Table 4).

#### Female genital tract

No CK1δ expression was detected in the oocytes (Figure 4D). CK1δ expression by granulosa cells was dependent on the maturation stage of the ovarian follicle. At the stage of the primary follicle, granulosa cells were strongly CK1δ positive. With advancing maturation, CK1δ expression gradually decreased until the antigen? was only faintly detectable in the epithelium of the mature follicle. Theka cells of mature follicles were CK1δ negative. The columnar epithelium lining the oviducts (Figure 4E) as well as the glandular epithelium of the endometrial mucosa (Figure 4F) displayed strong CK1δ immunoreactivity. A fraction of endometrial stroma cells expressed moderate levels of CK1δ.

#### Male genital tract

CK1δ expression was observed in male germ cells at various stages of differentiation. Moderate to strong staining characterised the spermatogonia located at the base of the seminiferous tubule. Upon differentiation, CK1δ expression was downregulated to reincrease slightly at the stage of secondary spermatocytes. Sertoli cells were weakly CK1δ positive (Figure 4G). In the interstitial space of the testis, strong CK1δ immunoreactivity of Leydig cells was conspicuous. Epithelial cells of the epididymis and the deferent duct expressed high amounts of CK1δ (Figure 4H) as well as the glandular epithelium of the prostate and the seminal vesicles (Figure 4I, J). In the seminal vesicles, CK1δ staining was predominantly baso-lateral, probably due to unstained secretion products located in the centre and the apical parts of the cell body (Figure 4J).

#### Immobile cells of mesenchymal origin

**Figure 5:**
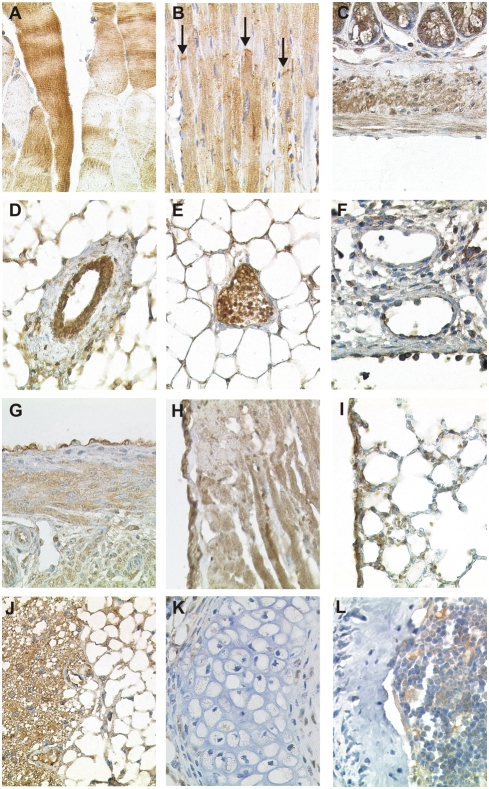
CK1δ expression in immobile cells of mesenchymal origin. Fixative: acid formalin, fixation by immersion. Peroxidase reaction, dye: DAB. CK1δ specific antiserum: NC10. The CK1δ immunostaining results of striated muscle cells of the skeletal system (A), the myocardium (B), and of smooth muscle cells of the intestinal wall (C); aterial blood vessel (D); venous blood vessel (E); lymphatic vessel (F); mesothelial cells of the peritoneum, (G) mesothelial cells of the pericardium (H), mesothelial cells of the pleura (I), adipocytes of white and brown fatty tissue (J), chondrocytes of the hyaline cartilage (K) and osteocytes (L) are shown. In B, arrows indicate intercalated discs of cardiomyocytes. Magnification: 200× (G, H, J), 400× (A–F, I, K, L).

Most striated muscle cells of the skeletal system and the myocardium were strongly CK1δ positive, only a few cells did not show any CK1δ immunoreactivity (Figure 5A, B). In skeletal muscle fibers, a heterogenous CK1δ staining pattern was observed with irregular band-like fields periodically arranged perpendicular to the long axis of the myofibril (Figure 5A). Such distribution was not apparent in cardiomyocytes that displayed a more diffuse CK1δ staining (Figure 5B). Notably, strong CK1δ staining frequently highlighted the intercalated discs in the myocardium. In contrast to striated muscle cells, CK1δ was only weakly expressed in smooth muscle cells in various organs, for example in the muscular layer of the intestinal wall (Figure 5C) and the lower urinary tract. However, smooth muscle cells in the media of arterial and venous blood vessels displayed practically no CK1δ staining (Figure 5D, E). CK1δ was also detected in the vascular endothelium. Endothelial cells of arteries showed strong CK1δ reactivity, whereas in veins, capillaries, lymphatics, and in high endothelial venules of secondary lymphatic tissue the endothelium was moderately to weakly stained or CK1δ negative (Figure 5D–F). Mesothelial cells at all sites, i.e. the peritoneum (Figure 5G), the pericardium (Figure 5H), and the pleura (Figure 5I) showed intermediate levels of CK1δ expression. In connective tissues, adipocytes of the univacuolar and polyvacuolar type were moderately CK1δ positive (Figure 5J). Fibroblasts and fibrocytes were negative or weakly positive (Figure 3M, N). CK1δ was neither expressed in chondrocytes of hyalin cartilage (Figure 5K) nor in osteocytes and osteoblasts (Figure 5L).

#### Hematopoietic and lymphoid organs

**Figure 6:**
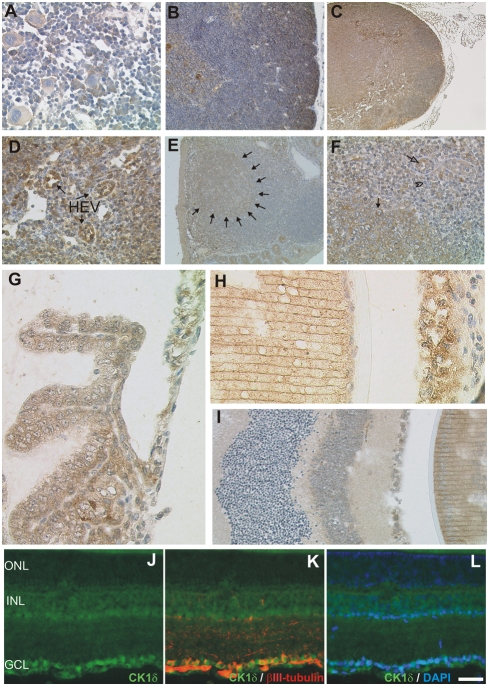
Localisation of CK1δ in hematopoetic and lymphoid organs and the eye. Fixative: acid formalin, fixation by immersion. Peroxidase reaction, dye: DAB. The CK1δ specific antibody NC10 was used to detect CK1δ in hematopoetic and lymphoid organs and in the eye. (A) bone marrow; (B) thymus; (C, D) lymph node; (E, F) cecal lymphoid follicle; (G) the eye ciliary body and iris; (H) lens and iris; (I) retina and lens. Immunohistochemical analysis of frozen retinal sections reveals localisation of CK1δ in retinal ganglion cells, which were specifically co-stained with an anti-βIII-tubulin antibody or DAPI (J–L). Magnification: 100× (B, C, E), 200× (D, F), 400× (A, G–L). ONL: outer nuclear layer; INL inner nuclear layer; GCL: retinal ganglion cell layer. Scale bar: 50 µm. Arrows in D indicate high endothelial venules (HEV), arrows in E delineate a B-follicle. In F closed arrows point to a lymphoid blast, the arrowhead points to a small resting lymphocyte and the open arrow indicates high endothelial venule.

Hematopoietic cells of the bone marrow were weakly to moderately CK1δ positive (Figure 6A). CK1δ expression seemed to correlate with immaturity, and down-modulation of the antigen was observed during intermediate maturation stages, including megakaryocytes. A more detailed assignment of CK1δ expression to specific differentiation steps and hematopoietic lineages could not be achieved on the CK1δ stained immunosections.

Immunohistochemistry revealed moderate CK1δ expression by juxtacortical thymocytes and a gradual downregulation of the antigen towards the cortico-medullary junction (Figure 6B). Thus, in the thymic cortex, loss of CK1δ staining seemed to be coincidental with T-cell maturation. In the medulla, CK1δ was expressed at moderate levels by a large fraction of cells. Owing to the heterogeneity of this microcompartment, it was not possible to positively identify T-lymphocytes in the thymic medulla on morphological grounds. The expression of CK1δ in the spleen has been specified in a previous report [35]. In the lymph node, substantial CK1δ staining was detected in the cortex, the paracortex, and the medulla (Figure 6C, D). All compartments contained CK1δ positive and CK1δ negative lymphocytes. CK1δ expression in primary B follicles was slightly lower than in the T-cell zone. However, secondary B follicles such as those in cecal patches of the gut-associated lymphoid tissue revealed a markedly elevated CK1δ staining in germinal centres, due to an intense expression by centroblasts (Figure 6E, F).

#### Eye

The squamous epithelium of the conjunctiva displayed strong CK1δ staining in the superficial layer while the basal layer was CK1δ negative. No CK1δ was detected in the sclera or the choroidea. Moderate CK1δ expression was seen in the epithelial part of the iris and the ciliary body (Figure 6G). The underlying stroma, including the sphincter muscle showed weak CK1δ staining. The capsule of the lens lacked any CK1δ expression (Figure 6H, I). However, the elongated epithelial fibers constituting the body of the lens were strongly CK1δ positive. Intermediate levels of CK1δ staining were observed in the cuboidal epithelium at the anterior surface of the lens (Figure 6H).

#### Retina

The ganglion cell layer and the inner nuclear layer, i.e., the third and second neuron of the retina were strongly or moderately CK1δ positive, respectively (Figure 6I). Double staining of CK1δ and βIII-tubulin localised the CK1δ positivity to the cell bodies of retinal ganglion cells (Figure 6J, K). CK1δ staining was also observed in the axons of retinal ganglion cells which are located in the retinal fiber layer. No CK1δ staining was seen in the photoreceptors, namely the outer nuclear layer.

#### Peripheral and central nervous system

**Figure 7:**
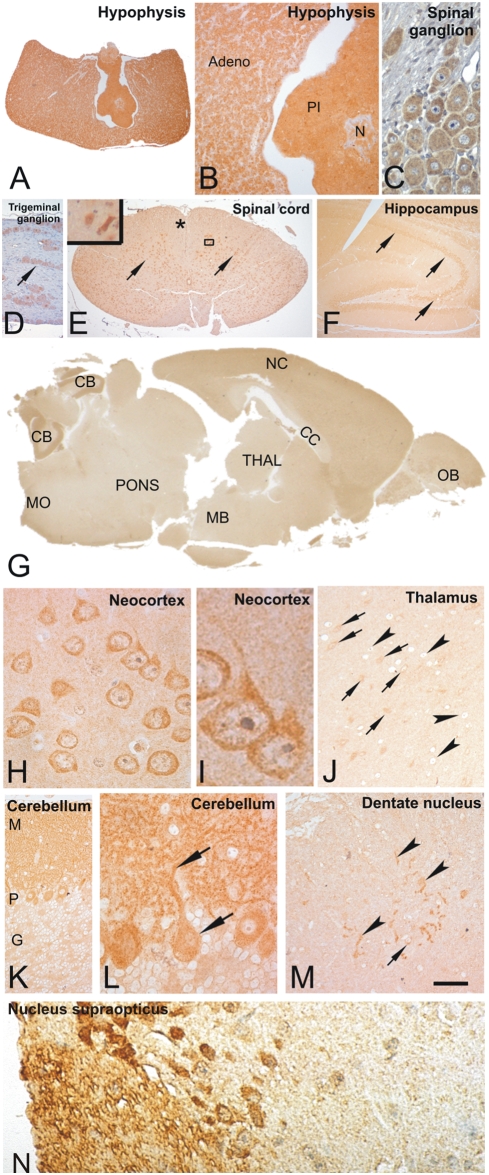
CK1δ expression in the nervous system. Fixative: acid formalin, fixation by immersion. Peroxidase reaction, dye: DAB. (A) CK1δ was strongly expressed in the hypophysis. (B) The high-power view shows a cytoplasmatic staining of the epithelial cells of the adenohypophysis (Adeno). The pars intermedia (PI) and the neurohypophysis (N) were also strongly marked. (C, D): The nerve cell perikarya of a spinal (C) and a trigeminal ganglion (D, arrow) exhibit CK1δ whereas the adjacent nerve fibers were not marked. (E) The spinal cord neurons were strongly marked (arrows) whereas the gray matter neuropil and the white matter only faintly exhibited CK1δ (asterix). The inset demonstrates the high power view into the boxed area and shows a marked cytoplasmic labelling. (F) In the hippocampal formation the neurons of the sectors CA1, CA2 and CA3 were strongly labelled (arrows). (G) Sagittal section of a mouse brain immunostained with the NC10 antibody directed against CK1δ. There are especially high levels of CK1δ detectable in all layers of the neocortex (NC), the olfactory bulb (OB), and the molecular and Purkinje-cell layer of the cerebellum (CB). The white matter as seen in the corpus callosum (CC) and the cerebellar white matter did not show high levels of CK1δ. Low levels of CK1δ were found in the thalamus (THAL), midbrain (MB), pons (P) and the medulla oblongata (MO). (H, I) At higher magnification neocortical neurons show a strong cytoplasmatic staining whereas the neuropil was weakly stained. There was no staining of glial cells detectable. (J) In the thalamus there was a very light staining of the neuropil. Some thalamic neurons exhibited CK1δ in the cytoplasm (arrows) whereas other neurons were not labelled (arrowheads). (K) In the cerebellum CK1δ stained the molecular layer (M) and the Purkinje cell layer (P). The granule cell layer neurons (G) were not marked. There was only a very light staining of the neuropil in this layer. (L) At the higher magnification levels it was evident that the CK1δ expression in the molecular layer was the result of the staining of the entire dendritic trees of the Purkinje cells. The arrows indicate a Purkinje cell with the apical dendrite positive for CK1δ. There was no labelling of Bergmann glia cells. (M) In the dentate nucleus of the cerebellum the neuropil was weakly stained whereas some neurons were strongly CK1δ positive (arrows). Neurites were also labelled in this nucleus (arrowheads). Hypothalamic nuclei showed varying levels of CK1δ. Some of these neurons, e.g. in the suprachiasmatic nulceus exhibited nuclear CK1δ. N. supraopticus (N). Calibration bar in L equals: A = 270 µm, B = 120 µm, C, H, L, N = 180 µm, D = 150 µm, E = 400 µm, E-inset = 55 µm F = 280 µm, G = 800 µm, I = 6.6 µm, J, M = 40 µm, K = 70 µm. CK1δ was stained with the antibodies NC10 (A–C, E–N) and abcam 10877 (D).

In the pituitary gland a significant cytoplasmic CK1δ expression was detected in cells of the adenohypophysis, the pars intermedia, and the neurohypophysis (Figure 7A, B).

The nerve cell perikarya of spinal and trigeminal ganglia exhibit CK1δ whereas the adjacent nerve fibers were not marked (Figure 7C, D). The spinal cord neurons were strongly marked whereas the grey matter neuropil and the white matter only faintly exhibited CK1δ (Figure 7E). There were high levels of CK1δ detectable in all layers of the neocortex, the olfactory bulb, and the molecular and Purkinje-cell layer of the cerebellum (Figure 7F, G, H). Neocortical neurons showed a strong cytoplasmic staining whereas the neuropil was softly stained (Figure 7I). There was no obvious staining of glial cells. In the hippocampal formation the neurons of the sectors CA1, CA3 and CA4 were strongly labelled (Figure 7E). In the cerebellum CK1δ stained the molecular layer and the Purkinje cell layer (Figure 7J, K). The granule cell layer neurons were not marked. There was only a very light staining of the neuropil in this layer. At the higher magnification levels it was evident that the expression of CK1δ in the molecular layer was the result of the staining of the entire dendritic trees of the Purkinje cells (Figure 7K). There was no labelling of Bergmann glia cells detectable. In the dentate nucleus of the cerebellum the neuropil was softly stained whereas some neurons were strongly CK1δ positive (Figure 7L). The white matter as seen in the corpus callosum and the cerebellar white matter did not show high levels of CK1δ. Low levels of CK1δ were found in the thalamus, midbrain, pons and the medulla oblongata. In the thalamus there was a very light staining of the neuropil. Some thalamic neurons exhibited CK1δ in the cytoplasm whereas other neurons were not labelled. Hypothalamic nuclei showed varying levels of CK1δ. Some of these neurons, e.g. in the suprachiasmatic nulceus, exhibited nuclear CK1δ (Figure 7M).

The expression of CK1δ in the spinal ganglia showed a strong immunoreactivity of the perikaryons (Figure 7N), whereas in the nerve fibres only a moderate to intermediate CK1δ staining intensity could be observed.

## Discussion

CK1δ plays an important role in the regulation of various cellular processes [2], [42]. However, many of its physiological functions are still unknown. The present study was carried out to determine the cell-specific tissue distribution of CK1δ in young adult BALB/c mice in order to obtain a better understanding of its biological functions.

A widespread distribution of CK1δ was detected in all frozen and paraffin embedded tissues although the expression levels differed among the analysed tissues and organs. However, the ubiquitous expression profile of CK1δ is consistent with previous RNA and protein expression profiling of CK1δ in different tissues [17], [26], [35], [37], [38], [43], [44]. Our results provide an anatomical backbone for future studies targeting cell-type specific functions of CK1δ. Since not much is known about cell-type specific functions of CK1δ, the discussion will be focused on those organs in which some cell-type specific functions of CK1δ have already been observed.

### Endocrine tissue

The strong granular cytoplasmic staining of CK1δ in the hypophysis, especially of the adenohypohysis and neurohypophysis, suggests important roles in the regulation of hormone secretion and storage. In this context it is worthwhile to notice that the CK1 family members α and δ have been localised to the synaptosome [45], [46]. They interact and phoshorylate several SNARE proteins, among them SV-2, syntaxin and snapin [47]–[49]. Phosphorylation of SNARE proteins has been suggested to influence their interaction with other proteins as well as vesicle transport and neurotransmitter release [48]–[55].

Moreover, the predominant distribution of CK1δ in endocrine tissues suggests critical roles in the regulation of hormone secretion. These findings are in line with the postulated role of CK1 family members in regulating vesicle budding and transport processes [1], [2], [19], [56], [57].

In the pancreas, the islets of Langerhans were stained much stronger than the exocrine portions including the duct system. However, islet cells differed in their degree of CK1δ expression. This could indicate that the expression of CK1δ is associated with the secretory phase of endocrine cells. In particular, it could be involved in the trafficking of secretory granules. On the other hand, this might reflect that different types of hormone secreting islet cells vary in their expression level of CK1δ. CK1δ expression could also depend on the activity status of the hormone regulating cells as hormone secretion relies on circadian periodicity. Accordingly, differences in hormone production and in the activity state of hormone producing cells might also explain the heterogeneous cytoplasmic staining of CK1δ of cells of the adrenal and pituitary glands.

CK1δ immunoreactivity was seen in the endocrine organs and in the disseminated endocrine cells of the gastrointestinal tract. In the testis, Leydig cells show a strong CK1δ positive staining suggesting a role of CK1δ in transport and release of testerone [2].

### Immune system

CK1δ expression was seen in all lymphatic tissues. CK1δ positive lymphocytes were detected in the PALS and the marginal zone of the splenic white pulp [35]. In secondary lymphatic organs of the intestine, a high CK1 expression was observed in lymphoblasts, indicating that CK1δ is induced in an antigen-specific manner. Thus, CK1δ could play a role in modulating the specific immune response.

### Central nervous system

CK1δ expression profiling in the brain indicated a broad distribution of CK1δ but differences in expression levels and subcellular localisation were detected. These results are in line with previous reports showing the expression of CK1δ RNA and protein in many different cerebral areas, e.g. in the striatum and neocortex, cerebrellum, hippocampus, thalamus, olfactory bulb and the midbrain region [17], [24], [43], [58].

An intensive CK1δ positivity was observed within the nucleus supraopticus which is a bilateral nucleus in the anterior hypothalamus involved in the regulation of the circadian rhythm. The neurons in this area showed, in addition to the cytoplasmic labelling, a strong nuclear CK1δ staining. These results are in line with previous observations [17] demonstrating that CK1δ modulates the stability, activity and nuclear entry of various “clock” proteins participating in the regulation of the circadian rhythm (reviewed in [27], [59]).

A strong CK1δ staining of the pericarya of neurons of the spinal cord, the hippocampus, the cerebrellum, the neocortex and the olfactory bulb were observed, whereas a heterogenous CK1δ positivity was seen in neurons of the thalamus. In each case a weaker CK1δ positivity of the neuropil was observed. Furthermore, a strong staining of the entire dendritic cells was seen. These observations might point to regulatory functions of CK1δ in neuronal signal transduction, especially in glutamatergic transmission pathways [43], [44]. The dendritic localisation of CK1δ might indicate a role of CK1δ in regulating neurite outgrowth, dendritic plasticity and stability by modulating the dynamic of both, the microtubule and the actin network. A possible function in regulating microtubule dynamics is further supported by the fact that CK1δ associates with and phosphorylates α/β-tubulin as well as microtubule associated proteins, like MAP1A, MAP4, tau, stathmin and APC ([2] and references therein, [61]). Deregulation of CK1δ has been shown to be associated with neurodegenerative diseases. CK1δ co-localises with granulovacuolar inclusions and tau-containing neurofibrillary tangles in Alzheimer's disease, Down syndrome, and Parkinson's disease. In Alzheimer's disease the co-localisation of CK1δ with tau points to a function for CK1δ in the abnormal processing/phosphorylation of tau [23], [24], [62]–[65]. The neurological breakdown in Parkinson's disease is predominantly correlated with the progressive degeneration of dopaminergic neurons. CK1δ has a regulatory role in dopaminergic neurotransmission, e. g. the CK1δ dependent activation of glutamate receptors results in high CK1 kinase activities in neostriatal neurons, leading to enhanced phosphorylation of DARPP-32 [43], [44].

### Reproductive organs

The pattern of CK1δ expression in the testis, i.e., a strong positivity in spermatogonia located at the base of the seminiferous tubules, might indicate a role in meiotic cell division. In fact, several CK1 isoforms have been shown to be important for accurate chromosome segregation during meiosis [20], [21], [66]. In addition, CK1δ might be required for the survival and development of germ cells. The ability of CK1δ to phosphorylate and/or to associate with motor proteins, AKAP proteins, MAPs, tubulin and actin binding proteins (reviewed in [2]) indicates its involvement in reorganising the cytoskeleton in spermatogonia. CK1δ might also be involved in the regulation of various processes in Sertoli cells, which provide structural and metabolic support to developing germ cells to which they are connected through multiple tight junctions. CK1δ has been shown to be associated with several tight junction proteins [67], [68] and could therefore be involved in regulating the dynamic reforming of tight junctions.

In conclusion, CK1δ is ubiquitously distributed in adult tissues, since phosphorylation events mediated by CK1δ could play an important role in regulating numerous tissue and organ specific processes. The dynamics of phosphorylation/dephosphorylation events may account for the extremely variable CK1δ expression pattern. In general, the expression level of CK1δ seems to depend on several parameters, such as the functional status, differentiation stage or gender. However, our profiling of CK1δ expression provides an anatomical backbone for future studies targeting cell-type specific functions of CK1δ in various tissues and organs.

## Materials and Methods

### Animals and Tissue Processing

BALB/c mice were bred in the Animal Facility of the Heinrich-Pette-Institute, Hamburg, and in the Animal Research Centre at the University of Ulm, Germany. All animal procedures conformed to institutional and European regulations concerning the protection of animals.

Tissue samples from 4 to 6 week old BALB/c female and male mice were immediately removed after killing and either shock-frozen or fixed by immersion in either 1% acetic acid in formalin, 10% buffered neutral formalin, zinc fixative [69], zinc-Formal-Fixx™ (Thermo Scientific, Fremont, CA, USA), NOTOX™ (Quartett, Berlin, Germany), Glyo-Fixx™ (Thermo Scientific, Fremont, CA,, USA), Bouin's or Carnoy's fixatives [70]. Alternatively, the animals were deeply anesthetized with Ketamin and fixed by cardiac perfusion with one of the following fixatives: 10% buffered neutral formalin, 1% acetic acid in formalin or Bouin's solution (see also Table 1). Bone tissue was decalcified with EDTA for several days at 4°C. Fixed tissues were then dehydrated in a graded ethanol series, cleared in methyl benzoate, and embedded in paraffin. Paraffin-embedded sections were cut at 3 µm and mounted on glass slides. Frozen tissue was embedded in Tissue-Tek (Sakura, Heppenheim, Germany). Sections (5–8 µm) were cut on a cryostat microtom (Leica, Bensheim, Germany), mounted on dry glass slides and fixed in 100% acetone for 10 min at 4°C.

### Primary antibodies

For IHC the CK1δ-specific polyclonal antisera NC10 (rabbit) [19], 108 (rabbit) [36] and ab10877 (goat) (abcam, Cambridge, GB) were used (see also Figure 2). The specificity of the rabbit antiserum 108 for IHC analysis was validated previously [36]. The specificity of NC10 was tested by immunoabsorption in immunohistochemistry using either the synthetic oligopeptide against which the antibody was raised, or an unrelated p53 specific oligopeptide (see Figure 1).

### Immunohistochemistry

#### Staining of paraffin sections

Staining procedures included deparaffinization in xylene, followed by rehydration via transfer through graded alcohols. To inhibit endogenous enzyme activity, Peroxidase Blocking Reagent (DAKO, Glostrup, Denmark) or Levamisole (DAKO, Glostrup, Denmark) were used. The sections were treated with different antigen retrieval solutions (Citra Plus (BioGenex, San Ramon, CA, USA), pH 6.03; AR-10 Solution (BioGenex, San Ramon, CA, USA), pH 10.7; Tris buffer, pH 7.3 as well as TUF solution, pH 5.7 (Kreatech, Amsterdam, Netherlands)) in a microwave oven, according to the manufacturer's instructions (see also Table 2). Sections were then incubated with one of the CK1δ specific antibodies (NC10, 1∶1200; 108, 1∶1200; ab10877, 1∶1600) at 4°C overnight. After washing in Tris-HCl buffer a horseradish peroxidase containing polymer conjugated anti-rabbit or anti-goat IgG antibody (N-Histofine^R^, Nichirei Corporation, Tokio, Japan), or alkaline phosphatase containing polymer coupled anti-rabbit IgG (N-Histofine^R^, Nichirei Corporation, Tokio, Japan) was applied at room temperature (RT) for 30 minutes. The enzymatic reaction was developed in a freshly prepared solution of 3, 3′-diaminobenzidine using DAKO Liquid DAB Substrate-Chromogen solution as a chromogen for horseradish peroxidase or with Newfuchsine Substrate-Chromogen (DAKO, Glostrup, Denmark) for alkaline phosphatase. The sections were then counterstained with hematoxylin and permanently mounted in Entellan (Merck, Darmstadt, Germany). Positive and negative controls were included for each case. As a negative control the primary antiserum was omitted and substituted with Tris-HCl buffer.

#### Staining of frozen sections

Frozen sections were quickly rehydrated in Tris-HCl buffer. Endogenous enzyme activity was blocked as described above. Sections were then incubated with either NC10 (1∶400), 108 (1∶400) or ab10877 (1∶200) for 40 minutes at RT. Slides were washed in Tris-HCl buffer and the DAB or Newfuchsine reaction performed as described above. Next, sections were counterstained with hematoxylin.

### Immunofluorescence analysis of frozen tissue

Eyes separated from connective tissue after perfusion fixation with PBS containing 14% paraformaldehyde were post-fixed for several hours, transferred to 30% sucrose overnight (4°C) and embedded in Tissue-Tek (Sakura, Heppenheim, Germany). Cyrosections were prepared as described above. Sections were labelled with monoclonal antibodies against βIII-tubulin (1∶2000, TUJ-1, Babco, Richmond, CA, USA) and against CK1δ (NC10, 1∶200). Secondary antibodies included anti-mouse IgG, anti-rabbit IgG and anti-sheep IgG antibodies conjugated to Alexa Fluor 488 and Alexa Fluor 594 (1∶1,000; Molecular Probes, Paisley, UK). For nuclear staining, sections were incubated in a solution containing 4′,6-diamidino-2-phenylindol (DAPI) for 2 minutes. Fluorescently labelled sections were embedded in Moviol (Calbiochem, Darmstadt, Germany) and analysed under a fluorescent microscope (Axioplan2, Zeiss, Jena, Germany).

### Grading System

Sections were graded with regard to intensity of the CK1δ specific staining. Intensity levels of the CK1δ specific staining were graded as − negative, + weak, ++ moderate, or +++ strong. Slash points to simultaneous expression of different intensities, e.g., −/++ indicates negative and moderately positive staining in one cell type or brain region.
